# Accounting for Population Stratification in Practice: A Comparison of the Main Strategies Dedicated to Genome-Wide Association Studies

**DOI:** 10.1371/journal.pone.0028845

**Published:** 2011-12-21

**Authors:** Matthieu Bouaziz, Christophe Ambroise, Mickael Guedj

**Affiliations:** 1 Department of Biostatistics, Pharnext, Paris, France; 2 Statistics and Genome Laboratory, University of Evry Val d'Essonne, UMR CNRS 8071 - USC INRA, Evry, France; Aarhus University, Denmark

## Abstract

Genome-Wide Association Studies are powerful tools to detect genetic variants associated with diseases. Their results have, however, been questioned, in part because of the bias induced by population stratification. This is a consequence of systematic differences in allele frequencies due to the difference in sample ancestries that can lead to both false positive or false negative findings. Many strategies are available to account for stratification but their performances differ, for instance according to the type of population structure, the disease susceptibility locus minor allele frequency, the degree of sampling imbalanced, or the sample size. We focus on the type of population structure and propose a comparison of the most commonly used methods to deal with stratification that are the Genomic Control, Principal Component based methods such as implemented in Eigenstrat, adjusted Regressions and Meta-Analyses strategies. Our assessment of the methods is based on a large simulation study, involving several scenarios corresponding to many types of population structures. We focused on both false positive rate and power to determine which methods perform the best. Our analysis showed that if there is no population structure, none of the tests led to a bias nor decreased the power except for the Meta-Analyses. When the population is stratified, adjusted Logistic Regressions and Eigenstrat are the best solutions to account for stratification even though only the Logistic Regressions are able to constantly maintain correct false positive rates. This study provides more details about these methods. Their advantages and limitations in different stratification scenarios are highlighted in order to propose practical guidelines to account for population stratification in Genome-Wide Association Studies.

## Introduction

Genome-wide association studies (GWAS) have become a widely used approach for gene mapping of complex diseases. With the development of high throughput genotyping technologies many markers are available to conduct these studies. The most common study design is the case-control design using unrelated individuals. The relevance of the results of such large scale genetic studies is however questioned. Indeed certain biases arise when conducting a GWAS, leading to false discoveries. As a consequence, only few associations are consistently and convincingly replicated [1]. There can be many causes to such spurious findings and non-replications [2]–[4]. It is broadly considered that failure to account for the bias induced by population stratification is one of them. This phenomenon occurs when the sampling has been made within non genetically homogeneous populations, i.e. there are systematic differences in allele frequencies due to ancestry and the baseline disease risk are different between the actual subpopulations. This can lead to finding spurious associations or to missing genuine ones [5]–[8]. Accounting for population stratification has nowadays become a necessary step in the conduct of a GWAS, especially with the development of very large studies such as the ones undertaken by international consortia. These studies indeed gather many cohorts of cases and controls, not always matched, with different ancestries.

The most used association test to detect an association is Armitage's Trend test. This test statistic follows a 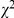 distribution under the null hypothesis of no association. In case of population stratification, this distribution is inflated and the test statistic follows a non-central 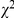 distribution. Several main approaches exist to account for population stratification in GWAS: Genomic Control [9], [10], Principal Component Analysis (PCA) based methods [11], [12], Regression models [4], [13], and Meta-Analyses. Genomic Control aims at correcting the Trend test statistic inflated null distribution by estimating an inflation factor, usually called 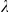, using many markers. In practice we usually consider that a 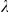 inferior to 1.05 indicates that there is no stratification [14]. The main assumption of this method is that the inflation factor is the same for all markers. PCA-based methods use markers to define continuous axes of variation, called principal components, that reduce the data to few variables containing most of the information about the genetic variability. These axes often relate the spatial distribution of the ancestries of the samples. Using such methods, Price et al. propose an association test to account for stratification. It is implemented in the software Eigenstrat [11]. In practice, it is also common to use the principal components to adjust the results of the classical association test to correct for stratification. These models are Adjusted Logistic Regression models and other adjustments such as on the discrete population labels can be used. Another possible approach to deal with population stratification is to conduct the analyses within subpopulations considered homogeneous and to combine the results with Meta-Analysis methods, such as Fisher's or Stouffer's *Z*-score methods [15]. It is also possible to use Structured Association methods to work around the stratification issues [16], [17]. These approaches aim at inferring the structure of the population using parametric models. The software Structure proposes this sort of approach [16]. A corresponding association test is available in the software Strat [18] but it is not as often utilized in practice. Note that other methods accounting for stratification, less used in practice, can be consulted in [19]–[27].

The potential of each approach to correct for population stratification depends actually on many factors such as the degree of stratification or the degree of sampling imbalance. This corresponds to situation where the proportions of cases and controls are not the same within the subpopulations. Three types of population structures can be highlighted [26]: discrete structures, admixed populations and hierarchical structures. Discrete structures correspond to cohorts composed of several discrete populations (e.g. African and Caucasian cohorts). Admixture structures pertain to cohort where the samples have admixed ancestries (e.g. African American). Hierarchical structures combine both discrete and admixture structures. The type of population structure is a very important parameter as it has a variable influence on all the methods, rendering them more or less efficient.

Many reviews and comparison articles looking at approaches to account for population stratification examined the potential of the methods [14], [28]–[32]. They focused on certain parameters affecting the stratification such as the sampling imbalance, the minor allele frequency of the disease susceptibility locus or the sample size. Most of them did not however exhaustively considered the different types of population structures. The study that we propose in this paper carefully analyzes this very parameter. We propose a comparison of the mainly used methods by considering a large panel of stratification scenarios corresponding to the different types of population structures. Our study differ from the recent comparison proposed in [32] by the methods considered and the type of simulations conducted. In our study numerous stratified datasets are simulated based on real data so that the structures of the population is well controlled and the data are similar to the ones used in real situations. We are interested in determining which methods tend to perform well, in term of false positive rate and power, under various situations. More precisely we aim at providing practical indications regarding which method(s) should be used with a given structure of the population as they account properly for the stratification bias. We address these questions for unstructured populations, admixed populations, discrete and hierarchical ones. Also, we propose a solution for situations where the sampling design has led to subpopulations only composed of cases or controls that haven't been genetically matched.

## Materials and Methods

First, we present the different methods that we decided to compare. Then we describe our process to simulate genetic data under various stratification scenarios. We provide precisions on the comparison strategy as well, i.e. how we estimated the statistical indicators that are the false positive rates and powers of the methods.

### A large panel of strategies compared

We decided to compare the performances of six broadly used strategies to account for stratification. First, we focused on the Genomic Control (GC) [9] and on the test proposed by Price et al. implemented in Eigenstrat (Eig) [11]. Then, we included adjusted Logistic Regressions (Reg). A large number of types of adjustments can be considered. We decided to focus on the mainly used in practice: adjustment on the five first principal components resulting from a PCA (Reg PCs), adjustment on the real population labels when this information is precisely known (Reg Real Pop) and adjustment on estimated population labels (Reg Est Pop). These latter labels were estimated using the method of Lee et al. [33]. We also studied one Meta-Analysis approach based on Fisher's score (Meta). Finally, we considered Armitage's Trend test, that does not account for stratification, as a reference to assess the level of stratification in the data.

Several additional adaptations of the Genomic Control, Regressions and Meta-Analysis where investigated as well. Since their results did not turned out to be significantly different from the original approaches, we will only consider them in the Discussion section. The six main methods investigated and their alternatives are detailed in Method S1, and a R script is available on demand.

### Simulation model

Our simulation model follows approaches previously used [34]–[36] and is based on the diplotype frequencies of real data sets. These frequencies are used as an empirical distribution of the range of possible diplotypes. Simulating this way leads to genetic patterns similar to those found in real data and therefore allows us to finely control the type of population structure. That way, we first simulate several datasets corresponding to the subpopulations of origin. Then we randomly mate each subpopulations and apply a genetic model to generate diseased and healthy samples. To simulate discrete subpopulations, the populations of origin are independently mated and for admixed populations we mate these populations with each other. The final subpopulations simulated are mixed together to produce a cohort of individuals with population structure. The type of population structure depends on the original datasets selected and the parameters of the model.

The genetic model is based on Wright's model [37] applied to a bi-allelic marker with susceptibility alleles *A* and *a*. Let 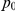, 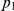 and 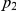 be the frequencies of genotypes *aa*, *aA* and *AA* defined by
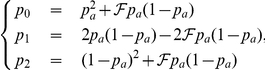
where 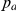 is the minor allele frequency of the SNP and 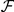 is the consanguinity coefficient that we consider null hereafter so that the Disease Susceptibility Locus (DSL) is under Hardy-Weinberg equilibrium.

We then want to compute the genotype frequencies of the DSL for cases and controls 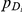 and 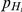, *i* = 0, 1 or 2, using the disease prevalence 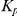, the penetrances 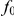, 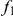 and 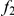 of the genotypes and the mode of inheritance of the disease. The main modes of inheritance can be defined by considering the relative risk 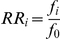, *i* = 1, 2 by
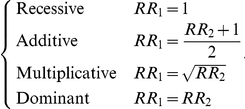
Using 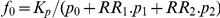, 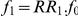 and 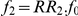 and the Bayes formulas we can easily derive the desired frequencies.
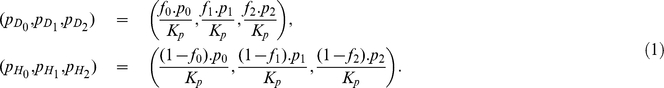
(1)


### Data sources and stratification scenarios

We simulated our data according the model described in the previous section and using the HapMap (http://hapmap.ncbi.nlm.nih.gov/downloads/genotypes/2010-08_phaseIIIII) populations. 5,500 SNPs, with minor allele frequencies higher than 5%, were randomly chosen in equal number on each of the non sexual chromosomes. We only considered SNPs present on an *Affymetrix GeneChip Human Mapping 500K* so that these SNPs are those commonly used in GWAS. Then, for each of our stratification scenario, some of the HapMap populations were used to simulate our final data with 5,500 SNPs and one DSL following an additive model and randomly located among the available loci.

We aimed at covering several situations as it may be harder to account for stratification with closely related populations than with very distant ones. Therefore, to get an exhaustive assessment of the strategies we considered several scenarios corresponding to different types of population structure: no structure, admixed populations, discrete structures with populations more or less genetically close, and a hierarchical structure. The proportions of cases and controls simulated are different in the subpopulations so that the design is not a simple random sampling. This and the differences between the populations ascertain that we induced and controlled a bias due to population stratification.

**Figure 1:**
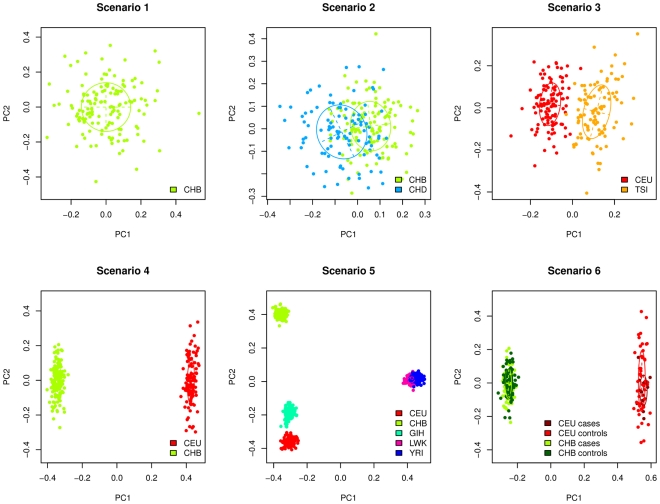
Population structures of the different scenarios. Samples are represented on the first two principal components (PCs) estimated on the genotype data.

The different scenarios that we considered are described hereafter and graphically represented in Figure 1. In addition, Table S1 gives the simulation parameters for these scenarios.

#### Scenario 1: One homogeneous population

With only one such population there is no stratification. The idea is to determine if the methods accounting for stratification are reliable when there are applied to a non-stratified population. Individuals from Han Chinese in Beijing, China (CHB) are used to simulate these data.

#### Scenario 2: Admixture

We considered an admixture of two originally close populations: Chinese in Metropolitan Denver, Colorado (CHD) and Han Chinese in Beijing, China (CHB) are used.

#### Scenario 3: Two fairly distant discrete populations

The two relatively distant discrete populations are Utah residents with Northern and Western European ancestry from the CEPH collection (CEU) and Toscans in Italy (TSI).

#### Scenario 4: Two very distant discrete populations

The two very distant discrete populations are Han Chinese in Beijing, China (CHB) and Utah residents with Northern and Western European ancestry from the CEPH collection (CEU).

#### Scenario 5: Hierarchical structure

The hierarchical structure is composed of five populations: Yoruba in Ibadan, Nigeria (YRI), Luhya in Webuye, Kenya (LWK), Han Chinese in Beijing, China (CHB), Gujarati Indians in Houston, Texas (GIH) and Utah residents with Northern and Western European ancestry from the CEPH collection (CEU).

#### Scenario 6: Varying proportions of cases/controls

This scenario uses the same populations as scenario 4 but with a varying proportion of cases between the two subpopulations. The proportion of controls is fixed and equal in the two populations while the proportion of cases is taken with a (*r*, 1 - *r*) ratio, with *r* varying. When this proportion is of 0 then all the cases are in the CEU population that is the less affected by the disease. When it is of 1 then all the cases are in the most affected population (CHB). Our goal is to observe the behavior of the methods in function of the degree of sampling imbalance and to look at whether they tend to perform well in the extreme case where all the cases come from only one of the populations. In this latter case, it is also of interest to determine if the best solution to account for population stratification is not to consider only the cohort composed of both cases and controls by excluding the samples that are not matched. The answer to this issue is particularly useful for large studies where controls with different ancestries are used to match the genotyped cases.

### Comparison strategy

We used a statistical framework to analyze the potential of the main approaches investigated that focuses on their false positive rates, also referred to as type-I-error rates, and powers. A statistical definition of these notions is provided in Method S2.

Note that population stratification is said to lead to spurious associations but also to mask true associations. This second effect is more tricky to observe but the statistical power can be useful to do so. As it corresponds to the proportion of SNPs that have been detected associated when they were, a loss of power between a situation with no stratification and a situation with stratification means that SNPs that used to be correctly detected in the first situation are no longer in the second. This corresponds to missing associations.

Both false positive rate and power can be expressed in function of the test statistic. However the distribution of this statistic is not always obvious so we prefer using the *p*-values instead. Thus the false positive rate becomes 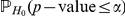 and the power 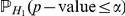. In our simulations, each dataset is simulated with one disease susceptibility locus, for which the degree of association is controlled, and 5,500 additional SNPs to assess the population structure. By placing ourselves under the null hypothesis, of no association, then under the alternative hypothesis, of association, we can respectively assess both false positive rate and power of the methods. To do so, we use a Monte-Carlo method and assess the same quantity
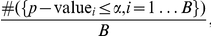
where # represents the cardinal function and *B* the number of simulated datasets.

All the DSL simulated, whether it is under the null hypothesis or the alternative, are differentiated. This implies that for all the population structures, one DSL is simulated per subpopulation. These DSL are excluded of the mating process the populations are then submitted to to reach the disired type of structure. That way, the properties of the DSL such as the relative risk are conserved whatever populaltion structure is simulated.

Note that only methods with equivalent false positive rate can be compared in term of power. This implies that a method with high power is no better than one with low power if the first one did not maintain a correct false positive rate.

We simulated data for several DSL relative risks ranging from 1 (no association) to 2.5 (strong association). For each relative risk a number of *B* = 2,000 datasets were simulated to get an accurate estimation of the statistical quality indicators. We genuinely estimated the indicators with this process as we controlled the degree of association through the simulation model. Note that there is an equivalence between the false positive rate and the power when the relative risk is of 1. A level 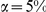 was chosen for all the tests. Data simulations and comparison of the strategies were performed using the software R (http://cran.r-project.org).

## Results

**Figure 2:**
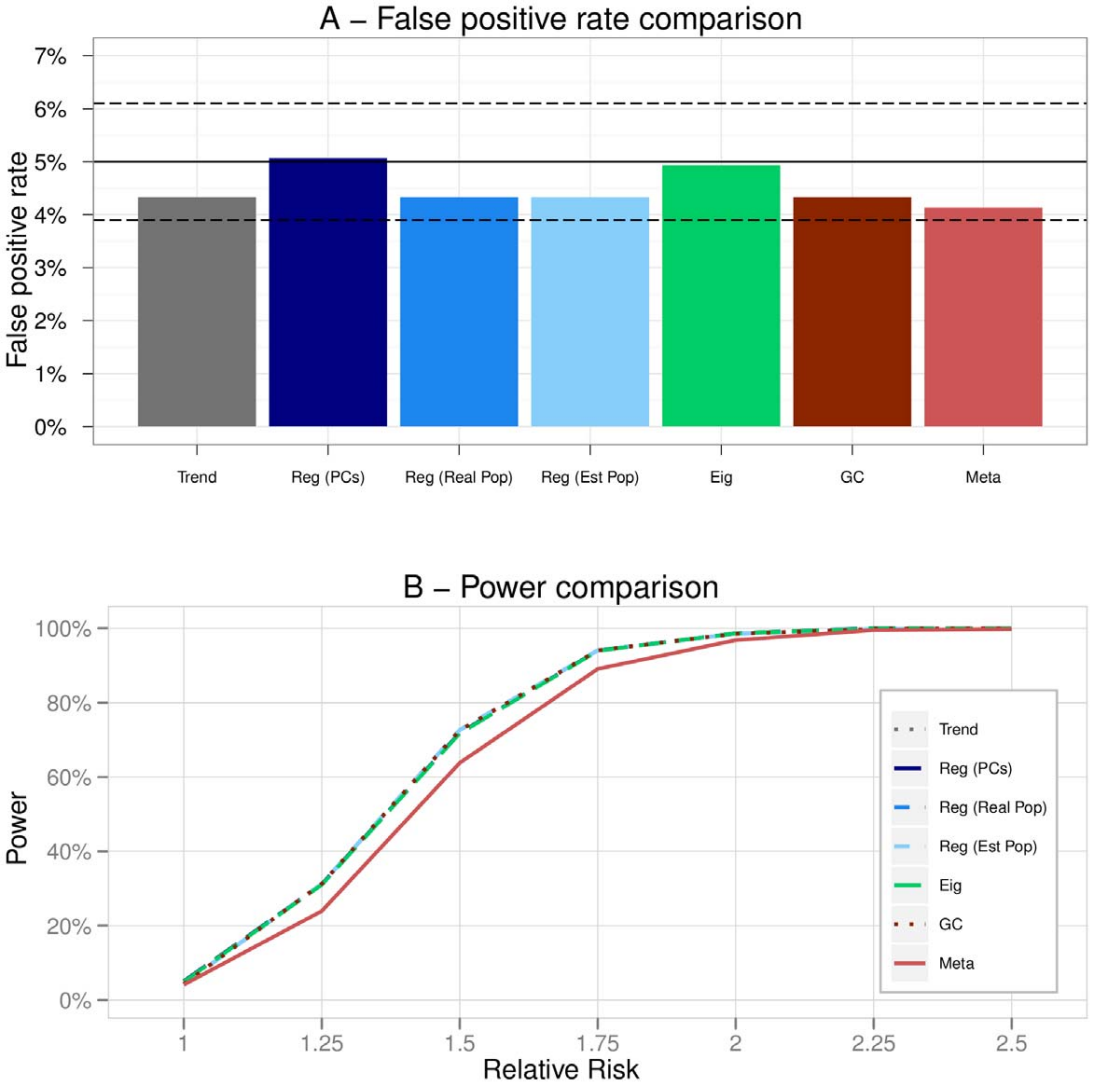
Scenario 1 (One homogeneous population). A - False positive rates of the methods. The plain black line represents the 5% level at which the tests were conducted. The dashed black lines are the 95% confidence intervals for this level. B - Powers of the methods in function of the increasing relative risk.

The results of the comparison are presented in this section for each scenario (Figures 2 to [Fig pone-0028845-g003] [Fig pone-0028845-g004] [Fig pone-0028845-g005] [Fig pone-0028845-g006] 7). Table S2 summarizes the estimations of 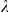 for the different scenarios. These estimations were conducted according to the methodology indicated in Method S1 by considering the median of Armitage's trend test statistics.

**Figure 3 pone-0028845-g003:**
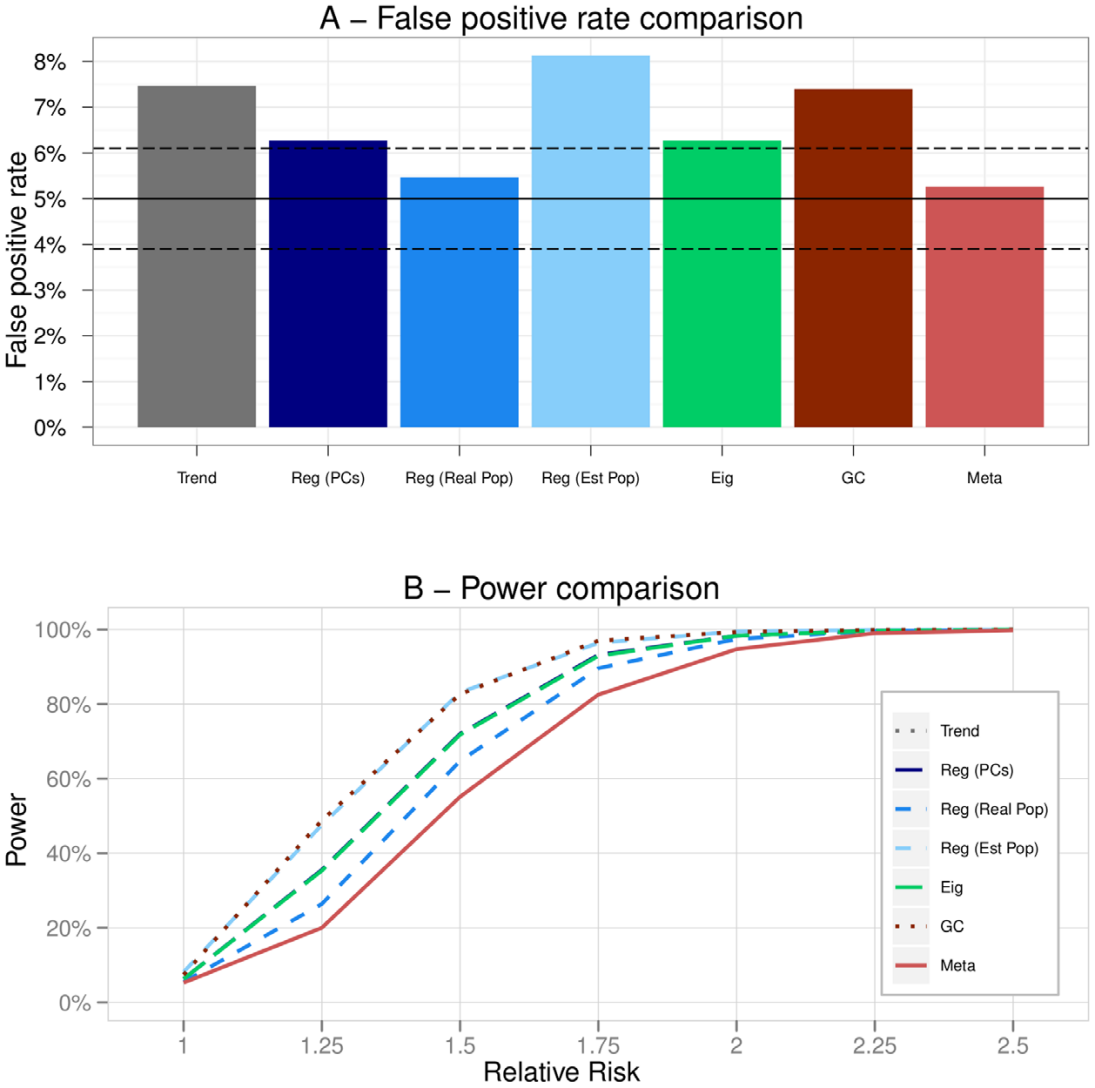
Scenario 2 (Admixture). A - False positive rates of the methods. The plain black line represents the 5% level at which the tests were conducted. The dashed black lines are the 95% confidence intervals for this level. B - Powers of the methods in function of the increasing relative risk.

**Figure 4 pone-0028845-g004:**
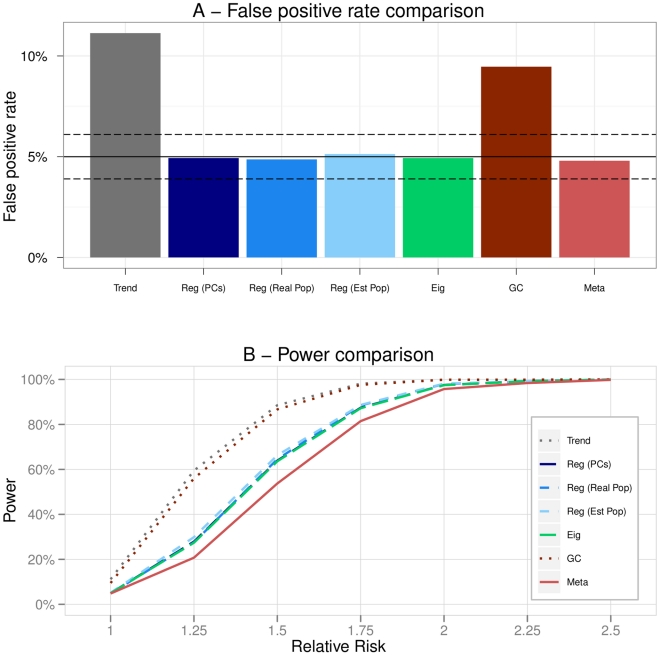
Scenario 3 (Two fairly distant discrete populations). A - False positive rates of the methods. The plain black line represents the 5% level at which the tests were conducted. The dashed black lines are the 95% confidence intervals for this level. B - Powers of the methods in function of the increasing relative risk.

**Figure 5 pone-0028845-g005:**
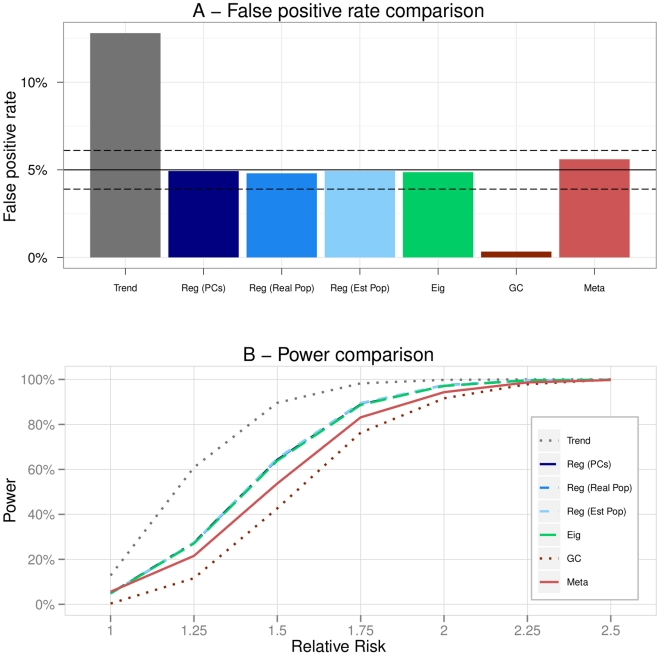
Scenario 4 (Two very distant discrete populations). A - False positive rates of the methods. The plain black line represents the 5% level at which the tests were conducted. The dashed black lines are the 95% confidence intervals for this level. B - Powers of the methods in function of the increasing relative risk.

**Figure 6 pone-0028845-g006:**
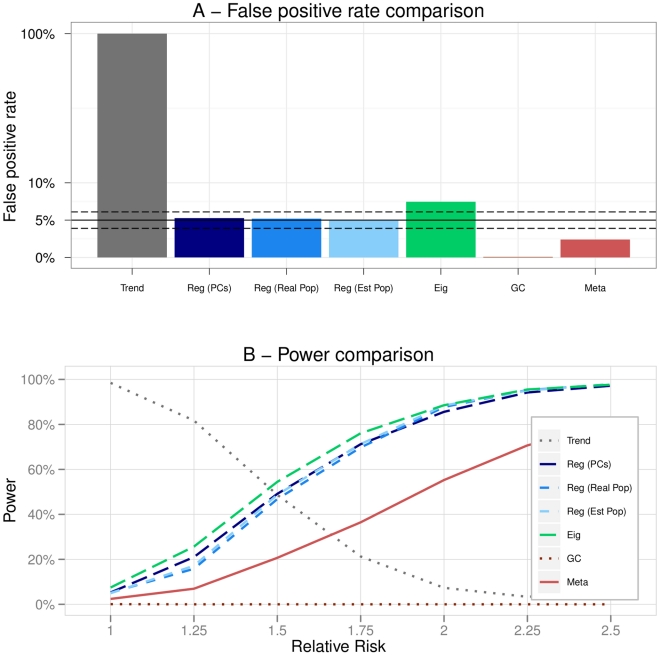
Scenario 5 (Hierarchical structure). A - False positive rates of the methods. The plain black line represents the 5% level at which the tests were conducted. The dashed black lines are the 95% confidence intervals for this level. B - Powers of the methods in function of the increasing relative risk.

**Figure 7:**
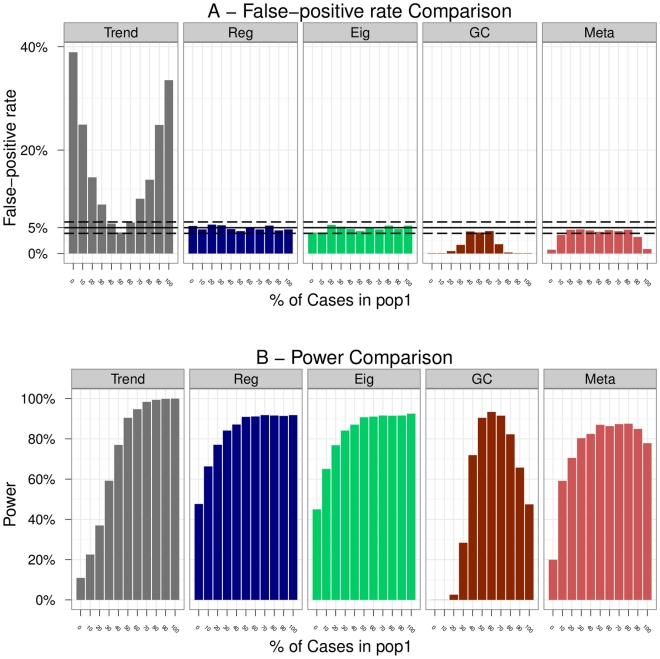
Scenario 6 (Varying proportions of cases/controls). A - False positive rates of the methods. The plain black line represents the 5% level at which the tests were conducted. The dashed black lines are the 95% confidence intervals for this level. B - Evolution of the power (with RR = 2) of the methods in function of the proportion of cases in pop1. Note that all the Regression methods being equivalent for this scenario, we summarize the results for these methods under the name ‘Reg’ only.

### Scenario 1: One homogeneous population

In the first scenario, with an unstructured population, the estimation of 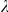 was 1.002 confirming that there was stratification. Figure 2-A presents the false positive rate of the methods. We noted that all of the methods had a correct false positive rate, lying within the 95% confidence bounds. Eigenstrat and Regressions adjusted on principal components (Reg PCs) were however the closest to the 5% level.

Figure 2-B provides the power curves of the different methods in function of the increasing relative risks. Powers of all the strategies were equivalent in this scenario except for Meta that was less powerful. One can note that there was no difference between an adjustment on a the real population labels and on the estimated ones. This was due to the fact that the population was so homogeneous that the clustering algorithm considered all samples to be in a unique population.

When there was no stratification, all the methods performed well and did not induce any bias. Besides, except for the Meta-Analysis, there was no loss of power when adjusting the results for stratification compared to the non-adjusted approach.

### Scenario 2: Admixture

This scenario corresponded to an admixture of two close populations. The estimation of 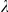 was 1.009 which meant that according to the Genomic Control there was almost no stratification.

However, one can observe that there was still a real bias induced by population stratification as the Trend test had a false positive rate significantly higher than 5% (Figure 3-A). This was also quite logically the case of the Genomic Control as the variance inflation factor was close to 1.

Eigenstrat and Regressions adjusted principal components (Reg PCs) had false positive rates reaching the upper bound of the confidence interval. Regressions adjusted on the estimated population labels (Reg Est Pop) led to a high number of false positive findings. This might have been due to the fact that the clustering algorithm used was not accurate enough to determine the correct population labels of the individuals in the case of an admixture.

The Regression adjusted on the real population labels (Reg Real Pop) and the Meta-Analysis had a false positive rate of almost 5%.

The analysis of the power curves (Figure 3-B) showed that the Trend test, the Genomic Control and the Regression adjusted on the estimated population labels (Reg Est Pop) had the highest powers. This was however due to the inflation of the false positive rate, also affecting the power, and therefore did not mean that these methods were more powerful. Eigenstrat and the Regression adjusted on the principal components were equivalent and outperformed the other methods in term of power. Regression adjusted on the real population labels (Reg Real Pop) and Meta were the less powerful method.

In an admixture scenario, so with a very fine population structure, only Eigenstrat, Reg (PCs) and Reg (Real pop) were correctly correcting for stratification.

### Scenario 3 and 4: Discrete structures

The third scenario corresponded to two populations closely related but that were differentiable. The estimated 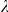 was 1.065 indicating a slight stratification according to the Genomic Control. Again the inflation factor was under-estimated as the false positive rate of GC was very high such as for the Trend test. All the other methods had a correct false positive rate (Figure 4-A).

On Figure 4-B, the power of Eigenstrat and the Regression methods were similar and higher than that of the Meta-Analysis.

In a situation where the populations were quite close it appeared that Eigenstrat and Regression based methods were the best solutions to account for stratification.

In scenario 4, the estimation of 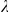 was 2.711 which denoted quite an important structure of the population. In such a situation , the Trend test was very biased and had a highly inflated false positive rate (Figure 5-A). On the other hand, the Genomic Control behaved differently and became too conservative. All Regression methods were equivalent and performed as well as Eigenstrat both in term of false positive rate and power. Again the Meta-Analysis was the less powerful strategy (Figure 5-B).

### Scenario 5: Hierarchical structure

Scenario 5 pertained to a more complex population structure. There were five populations and a hierarchical structure leading to an estimation of 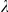 of 9.571. It was striking how the Trend test deviated from the 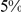 level by reaching almost 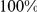 of false positive findings under the null assumption. On the contrary, the Genomic Control was very conservative due to the high value of 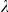. Eigenstrat had an inflated false positive rate and was no longer equivalent to the adjusted Regressions. In addition, we observed that Meta was too conservative in this scenario (Figure 6-A).

The Genomic Control was not powerful at all as it did not detected any association. Powers of all the Logistic Regressions were slightly smaller than that of Eigenstrat but this was due to the difference in false positive rates (Figure 6-B).

In such a situation, only Logistic Regressions were capable of keeping correct false positive rates while reaching good powers.

### Scenario 6: Varying proportions of cases/controls

The sixth scenario corresponded to the same population structure as the fourth but with a varying sampling design. Figure S1 presents the evolution of 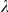 with the proportion of cases.

We observed that the Trend test had a correct false positive rate only when the sampling design was balanced between the two populations otherwise it was inflated. The opposite trend was noticeable for the Genomic Control (being quickly too conservative) and Meta. On the other hand, whatever the sampling design, Regressions and Eigenstrat globally maintained a correct false positive rate (Figure 7-A). When the sampling was very imbalanced however, Eigenstrat tended to deviate from the 5% level.

The analysis of the power (Figure 7-B) showed us that powers of Regressions and Eigenstrat were equivalent which confirmed the result that we previously found in scenario 4.

An interesting fact was to observe the loss of power of the Trend test between the extreme situations. This confirmed that population stratification can lead to missing genuine associations. Quite logically we also retrieved the fact that if individuals are sampled in a very affected population then the power was more important than in other cases.

It is quite common in GWAS to include patients having different ancestries than the original cohort. This can be done to get larger samples or to find controls corresponding to the typed cases. A larger sample size implies a gain in power, however if ancestries are different, population stratification could generate a bias reducing the power. If one of the group of patients with a different ancestry than the rest of the cohort is only composed of controls (or cases), one practical question often discussed is whether it is better to exclude this cohort of the study or to keep it and account for stratification.

We answered this question by comparing the powers of the methods when all the patients were kept and when only the cohort composed of both cases and controls was kept. We focused only on Regressions and Eigenstrat that were the methods able to correctly correct for stratification. Whether all the cases were in the most affected or in the less affected population, we observed that the powers were the same whether the cohort composed of controls only was excluded or not. The power was not more important with more samples because of the bias due to stratification. However this bias was taken into account by the two methods so that it was not necessary to exclude a part of the patients (Figure S2).

### Computational considerations

In term of execution time, the investigated methods are relatively equivalent. The Genomic Control is relatively fast as it imply to test two times each SNP. Adjusted Regressions and Eigenstrat are quite equivalent when principal components are used to adjust the results. The necessary time to adjust on estimated population labels depends on the algorithm used to infer the population structure and can be quite fast or very time consuming.

It has been pointed out that Linear Regression can be a practical alternative to Logistic Regression as it is computationally faster, especially when there are covariates included in the models [38]. We analyzed this method as well in our study (data not shown). Linear and Logistic Regression methods seemed to be perfectly equivalent in most of the scenarios, however it appeared that the use of a dichotomous outcome such as the disease status in the Linear Regression is no longer a viable options in hierarchical populations (scenario 5). We therefore recommend to keep using the Logistic Regression instead.

## Discussion

Genome-Wide association studies are more and more used. The problem of population stratification is however a serious shortcoming for these studies, raising doubts about their findings. To counteract this effect many approaches have been developed to account for stratification but it is not always clear in which situations they should be applied. Several articles have been published studying the performances of the different methods when some parameters influencing the stratification bias such as the minor allele frequency of the susceptibility locus, the degree of sampling imbalanced, the number of markers or the sample size vary [14], [28]–[32]. We have decided to focus here on a parameter that has not been studied in depth and is yet quite important that is the type of population structure itself. Indeed, one can wonder whether it is a good thing to adjust for stratification when there is no structure of the population, or whether reducing the bias is easier with distant or close populations. Also the relative performances of the most commonly used approach under these scenarios may vary differently. We compared these approaches through simulation studies by considering several scenarios of population structures. A particularity of our study is that to do so, we used a robust simulation model that is based on real diplotype data so that we simulated datasets similar to the ones used in real situations.

We first determined that if there is no structure in the population, all of the studied methods correcting for stratification performed well both in term of false positive rate and power reflecting trends previously reported [21], [22], [32]. Given this result and since it is quite difficult to be entirely sure that the population is sufficiently homogeneous, we recommend to always apply a correction for the stratification bias.

Concerning the type of population structure, our study also pointed out the fact that as soon as there is an admixture in the structure (scenarios 2 and 5) then it is more delicate to correct the bias than with discrete populations.

We then highlighted methods that did not provide a good correction for stratification. First, we showed that the Genomic Control failed to properly account for stratification in most of the situations. An interesting observation is that this method was not always affected in the same manner by the stratification. For genetically close populations the variance inflation factor 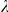 was not a good indicator of the stratification level as it indicated almost no structure. This means that the Genomic Control was anti-conservative. On the other hand, with relatively distant populations, this factor was overestimated, and therefore the false positive rate below the 5% level, rendering the Genomic Control a too conservative method. We therefore confirm the conservativeness of the Genomic Control reported in many situations [28], [29], [39]. We also studied an alternative version of the Genomic Control, where the estimation of 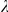 was based on the mean of the test statistics and instead of on the median. This version provided the same results as the one we presented in this paper.

Second, in most of the scenarios we noted than the Meta-Analysis method was less powerful than the other alternatives. If it is however required to use a Meta-Analysis method then Fisher's method appeared as the best option. Indeed, we compared the Fisher and the *Z*-score methods and found that Fisher's always had a correct false positive rate and a better power.

We therefore do not recommend the use of the Genomic Control and Meta-Analyses methods to get a proper correction for stratification.

Note that it was not possible in our study to include the test implemented in the software Strat which is based on the results of Structure as the underlying algorithms are computationally very intensive [14], [29]. This rendered difficult to compare the test to the other methods in a robust manner. Even though it has been shown that Strat can provide a reasonable correction for stratification [29], its high computational cost and complexity would lead us not to consider this test to account for stratification when conducting a GWAS.

Our results pointed out that the test implemented in the software Eigenstrat is a good solution to account for stratification with admixed or discrete structure which confirms the findings of [29], [32], [40]. On the other hand, with a hierarchical structure (scenario 5), we found that Eigenstrat had a false positive rate deviating from the 5% level which has been reported by previous studies [26], [32]. In the recent comparison study [32], no hierarchical structure was investigated however the inflated false positive rate of Eigenstrat was reported for stratification scenarios including several populations or admixtures. Given that Regressions were able to correct the bias in a satisfactory way in this scenario it implies that Eigenstrat and the Logistic Regressions adjusted on the principal components are not always equivalent. This results is also outlined in [32].

Note that we included 5 principal components for the regression adjustements and Eigenstrat. It is also of interest to look at the quality of the corrections if more or less components are considered. Additional simulations considering 1, 2, 5, 10, 20 or 50 components were conducted. They show that for a structure relatively simple to infer (scenario 4), the number of principal components included in the models do not have an influence on the adjustements. Both the logistic regression and Eigenstrat have correct false positive rates and comparable powers (Figure S3). When the structure of the population is more complex (scenario 5), more components are needed to keep a reasonable false positive rate (Figure S4). The logistic regression has an inflated false positive rate if only one component is used and a better power if more than two components are used. It is interesting to note that Eigenstrat has a false positive rate that is no longer outside of the condifence interval for the 5% level when many components are used (more than ten in our simulations). This however goes along with a consequent loss of power. This might be the reason why Price et al. advised a default number of ten components when using this method [11]. Logistic regression is therefore more stable than Eigenstrat to the number of principal components used.

We also showed that the most efficient methods to account for stratification make use of Logistic Regressions. In all of the situations studied here these methods were able to maintain a proper false positive rate and provided a good power to detect associations.

Concerning the different types of adjustments, one has to note that the Regressions adjusted on the real population labels may not be applicable in every situations since an accurate information about the sample ancestries is not always available. If the information available is not accurate enough then estimated labels may be more informative about the homogeneous subgroups and should be used instead [41].

We also investigated alternative Regression based approaches that were not discussed in the results section but that are closely related to the main approaches we presented. First, we investigated another method combining the use of estimated population labels and principal components to adjust the association test [40]. This method was not different than using only the principal components in our data. The rational invoked by Li et al. to use both adjustments to respectively account for discrete and admixed populations is however pertinent making this method a reasonable option when the population labels can be accurately estimated. In addition, we investigated the use of estimated population probabilities instead of the discrete labels which showed that both methods are equivalent.

Another important question is how the methods behave when the sampling proportions become more imbalanced between the subpopulations. We addressed this question in the sixth scenario that highlighted the fact that Regressions and Eigenstrat were the methods capable of correcting for stratification even with very imbalanced samplings. In the extreme cases where all the cases are from one population only, we observed that considering only the cohort composed of both cases and controls by excluding the cohort with controls only was as powerful as considering all the samples. This highlights that adjusted Logistic Regressions and Eigenstrat are performing well enough so that they can deal with extreme sampling within subpopulations.

New sequencing methods allow to focus on DSL with very low minor allele frequency (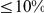). In order to determine the quality of the methods to account for stratification with such DSL we simulated additional datasets corresponding to the scenario 4 and 5 (Figure S5 and S6 respectively). It appears that the approaches considered have the same behavior than with more important minor allele fresuencies but they all experience a loss of power. This loss of power is expected when testing a non-stratified association with low minor allele frequency and our results confirm the findings of [29] that is it still the case with stratification.

Finally, we expect that when the number of SNPs available in a study increases, the information about the structure of the populations and therefore the quality of the corrections of all the methods also increase. This is confirmed by the comparisons conducted in [29], 32 considered more than 10,000 SNPs. When a certain amount of SNPs is reached, usually tens of thousands, the information provided by additional SNPs becomes redundant (e.g because of linkage disequilibrium) and the corrections are no longer better. Also, when the amount of SNPs included is not important enough, usually less than a couple of hundreds, the methods are not provided with enough information to properly account for stratification.

To conclude, we summarize the performances of the main methods studied in this paper for all the types of population structure Table 1. Given the results we presented, we recommend to use, whatever the population structure, an adjusted Logistic Regression model. The adjustment on the principal components is the more advantageous as it always leads to a correction of the bias. Moreover, principal component analysis can always be applied to the genetic data without any previous knowledge on the structure. If one has some accurate information on sample labels, then a joint adjustment with the principal components should provide an even better correction.

**Table 1:** Summary table.

Method	Type of correction	No Strat		Admixture		Discrete Strat		Hierarchical	
		FP	Power	FP	Power	FP	Power	FP	Power
Trend	None	++	++	−	.	−	.	−	.
Reg (PCs)	Continuous	++	++	+	++	++	++	++	++
Reg (Real Pop)	Discrete	++	++	++	+	++	++	++	++
Reg (Est pop)	Discrete	++	++	−	.	++	++	++	++
Eigenstrat	Continuous	++	++	+	++	++	++	−	.
GC	Continuous	++	++	−	.	−	.	−	.
Meta	Discrete	++	+	++	+	++	+	−	.

This table summarizes the results of our study in terms of false positive rate and power. A ‘++’ implies a very good performance, a ‘+’ a good performance, a ‘−’ a bad performance and a ‘.’ that it was not possible to assess a comparable power given that the false positive rate was not correct.

FP: False positive rate.

## Supporting Information

Method S1 A detailed description of all the statistical tests investigated in the study.
(PDF)

Method S2 Details about the notions of false positive rate and power.
(PDF)

Figure S1Evolution of 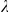 for Scenario 6. Representation of 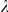 estimated with GC in function of the proportion of cases in pop1.(PDF)

Figure S2 Power comparison for scenario 6 with one or two cohorts. The powers of Reg and Eigenstrat (EIG) are represented when keeping the two populations (Both) or when excluding the population with only controls (One). On the left hand all the cases are in pop2 (less affected by the disease) and on the right hand all the cases are in pop1 (more affected by the disease).
(PDF)

Figure S3 False positive rate and power comparison of principal component based methods with varying number of components included in the models in scenario 4.
(PDF)

Figure S4 False positive rate and power comparison of principal component based methods with varying number of components included in the models in scenario 5.
(PDF)

Figure S5 False positive rate and power comparison of the methods with low minor allele frequency for the scenario 4.
(PDF)

Figure S6 False positive rate and power comparison of the methods with low minor allele frequency for the scenario 5.
(PDF)

Table S1 Simulation parameters for the stratification scenarios.
(PDF)

Table S2Estimated 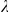 for the different scenarios.(PDF)
