# Supplementary material for: Accounting for Population Stratification in Practice: A Comparison of the Main Strategies Dedicated to Genome-Wide Association Studies
Source: PLoS One. 2011 Dec 21;6(12):e28845. doi: 10.1371/journal.pone.0028845 (PMC3244428; doi:10.1371/journal.pone.0028845)
Supplement: Method S1 — A detailed description of all the statistical tests investigated in the study. (PDF) [file pone.0028845.s001.pdf]

Method S1

These Supporting Information describe the methodologies of all the statistical tests investigated. We consider the binary variable Y to be the patients' status, and X the SNP tested. X is usually coded 0, 1 or 2, indicating the number of minor alleles present at the corresponding locus. Let also N be the number of samples used for the tests. We consider the null and alternative hypotheses of all tests to be

H_0: There is no association between Y and X,

H_1: There is an association between Y and X.

Armitage's Trend test

Armitage's Trend test is equivalent to an unadjusted Logistic Regression. This association test corresponds to testing the nullity of the β coefficient of the logistic model

$$\text{logit}(\mathbb{P}(Y = 1)) = \alpha + \beta X, \tag{1}$$

where α and β are parameters to be estimated. The test statistic Z of this test follows a $\chi^2(1)$ under the null hypothesis.

Genomic Control

Genomic Control [1, 2] aims at correcting the inflated null distribution of the test statistic of Armitage's Trend test using an inflation factor λ. There are several ways to estimate this factor using many loci. We considered two of them using the inflated values of the Trend test statistics $Z_1, \ldots, Z_p$ of p markers assumed non-associated with the disease (i.e. all the markers available in our simulations except the DSL). One can estimate

$$\lambda_{med} = \frac{\text{median}(Z_1, ..., Z_p)}{0.456}$$

and

$$\lambda_{\text{mean}} = \text{mean}(Z_1, ..., Z_p).$$

These factors λ are then use to correct the distribution of the test statistic Z of the SNP tested for association so that the new distribution Z/λ follows a $\chi^2(1)$.

The standard version of the Genomic Control, using the factor λ_{med}, is implemented in `Eigensoft` [1] or `Plink`[2]

Eigenstrat

The test proposed by Price et al. [3] to test the association between the SNP X and the phenotype Y uses the result of a principal component analysis (PCA) applied to many markers used to assess the axes of variation describing the structure of the population. Both the SNP and the phenotype are projected

[1] `http://genepath.med.harvard.edu/~reich/Software.htm`

[2] `http://pngu.mgh.harvard.edu/~purcell/Plink/`

on the selected axes of variation to provide adjusted values, respectively X_{adj} and Y_{adj}, that are corrected for stratification. The test statistic is

$$(N-1-k) \times corr(X_{adj}, Y_{adj})^2,$$

where N is the number of samples and k the number of retained principal components. This statistic follows a $\chi^2(1)$ distribution under the null hypothesis.

Adjusted Logistic Regressions

Several adjustments of the Logistic Regression models are possible by adding covariates to the model (1). The model becomes

$$\text{logit}(\mathbb{P}(Y=1)) = \alpha + \beta X + \text{Covariate(s)}.$$

Adjustment on the principal components: Reg (PCs)

The adjustment on the principal components uses the results of the PCA applied to SNPs data such as described in the previous section. Principal components $PC_1, \ldots, PC_k$ are added to the logistic model as covariates.

Adjustment on population labels: Reg (Real Pop) and Reg (Est Pop)

The adjustment on discrete population labels corresponds to adding a discrete variable $(a_i)_{1\leqslant i\leqslant N}$ to the model. This variable indicates the population of origin of each sample. It can relate to the real populations of origin for the case of Reg (Real Pop) or the estimated ones for Reg (Est Pop).

Many clustering algorithms are available to estimate the population labels including Hierarchical clustering, `Structure` [4], or other clustering algorithms that can be applied to the principal components [5].

We decided to consider in the paper population labels estimated with Gaussian Mixture models (GMM) applied to the principal components $PC_1, \ldots, PC_k$ such as proposed by Lee et al [5]. The `R` package `Mclust`, conducting a clustering estimation using GMM, was used to compute the vector $(a_i)_{1\leqslant i\leqslant n}$ of estimated population labels.

Whatever the type of population structure considered it was possible to estimate discrete population labels with the clustering algorithm. When using real population labels, the discrete information was only available for discrete structures. Therefore, we divided for the admixed structure that an individual with most of its genome coming from one of the subpopulations belonged to this subpopulation to define discrete real population labels.

Alternative adjustments

The adjustment on the clustering probabilities corresponds to adding as much covariates as there are estimated subpopulations using the method of Lee et al. described above. Each of these covariates corresponds to the probability of the samples to belonged to each of the subpopulations.

The joint adjustment on principal components and population labels is conducted by including both set of variables as covariates in the logistic model.

The Linear Regression models considered can be adjusted on the same covariates as the Logistic ones. They also involves the estimation of one parameter to determine whether there is an association or not.

Meta-analyses

Meta-analysis methods consist in calculating SNP-disease association p-values $(p_i)_{1\leq i\leq K}$ using Armitage's Trend test in each of the supposed K homogeneous subpopulations and combining them afterwards.

It is implied that one needs some knowledge about the homogeneous subpopulations, i.e. about the populations labels. Real ones or estimated ones can be used to conduct meta-analyses.

Fisher's method

Fisher's statistic $\sum_{i=1}^{K} log(p_i)$ follows a $\chi^2(2K)$ distribution under the null hypothesis.

Stoufler Z-score method

Stoufler's Z-score statistic is $\sum_{i=1}^{K} F^{-1}(1-p_i)$, where F is the standard normal cumulative distribution and follows a standard normal distribution under the null Hypothesis.

References

1. Devlin B, Roeder K (1999) Genomic control for association studies. Biometrics 55: 997–1004.
2. Reich DE, Goldstein DB (2001) Detecting association in a case-control study while correcting for population stratification. Genet Epidemiol 20: 4–16.
3. Price AL, Patterson NJ, Plenge RM, Weinblatt ME, Shadick NA, et al. (2006) Principal components analysis corrects for stratification in genome-wide association studies. Nat Genet 38: 904–909.
4. Pritchard JK, Stephens M, Donnelly P (2000) Inference of population structure using multilocus genotype data. Genetics 155: 945–959.
5. Lee C, Abdool A, Huang CH (2009) Pca-based population structure inference with generic clustering algorithms. BMC Bioinformatics 10 Suppl 1: S73.
