# Supplementary material for: Accounting for Population Stratification in Practice: A Comparison of the Main Strategies Dedicated to Genome-Wide Association Studies
Source: PLoS One. 2011 Dec 21;6(12):e28845. doi: 10.1371/journal.pone.0028845 (PMC3244428; doi:10.1371/journal.pone.0028845)
Supplement: Method S2 — Details about the notions of false positive rate and power. (PDF) [file pone.0028845.s002.pdf]

Method S2

We provide in this supplementary the basic definition of the notion of false-positive rate, or type-I-error rate, and power. Considering a test looking at a null hypothesis H_0 and an alternative hypothesis H_1 under the significance level α we can draw the following table describing the possible outcomes.

	H_0 is accepted	H_0 is rejected
H_0 is true	True Negative $(1-\alpha)$	False Positive / Type-I error rate (α)
H_0 is false	False Negative / Type-II error rate (β)	True Positive / Power $(1-\beta)$

Definition: The false positive rate is the probability of rejecting the null hypothesis when this hypothesis is true, i.e. the probability of detecting a SNP associated with the disease when it is not the case. It corresponds to $\mathbb{P}_{H_0}(\text{rejecting } H_0)$. All the tests are conducted at the same level α which means that we expect a false positive rate of α as well.

Definition: The statistical power is defined as the probability of rejecting the null hypothesis when the alternative hypothesis is true, i.e. the probability of detecting an association when there is actually one. Formally speaking, the statistical power corresponds to $\mathbb{P}_{H_1}(\text{rejecting } H_0)$ and is function of the test significance level α.
