# Supplementary figures and images for: Accounting for Population Stratification in Practice: A Comparison of the Main Strategies Dedicated to Genome-Wide Association Studies
Source: PLoS One. 2011 Dec 21;6(12):e28845. doi: 10.1371/journal.pone.0028845 (PMC3244428; doi:10.1371/journal.pone.0028845)

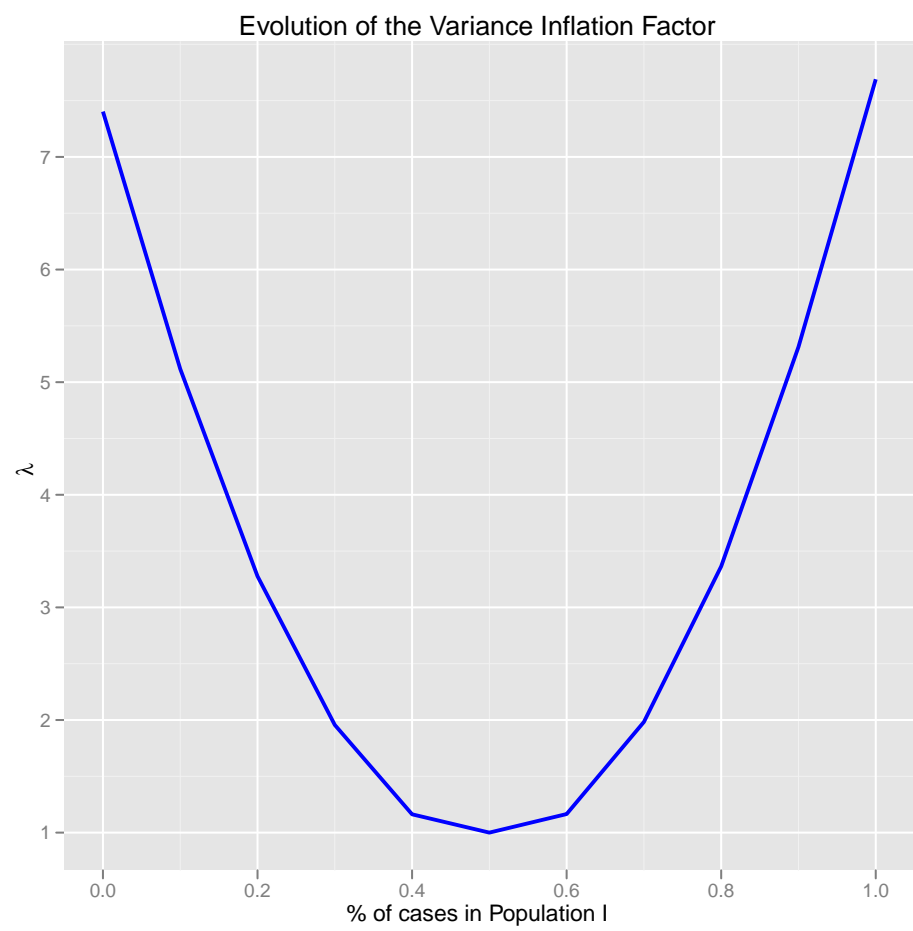

Supplement: Figure S1 — Evolution of for Scenario 6. Representation of estimated with GC in function of the proportion of cases in pop1. (PDF) [file pone.0028845.s003.pdf]

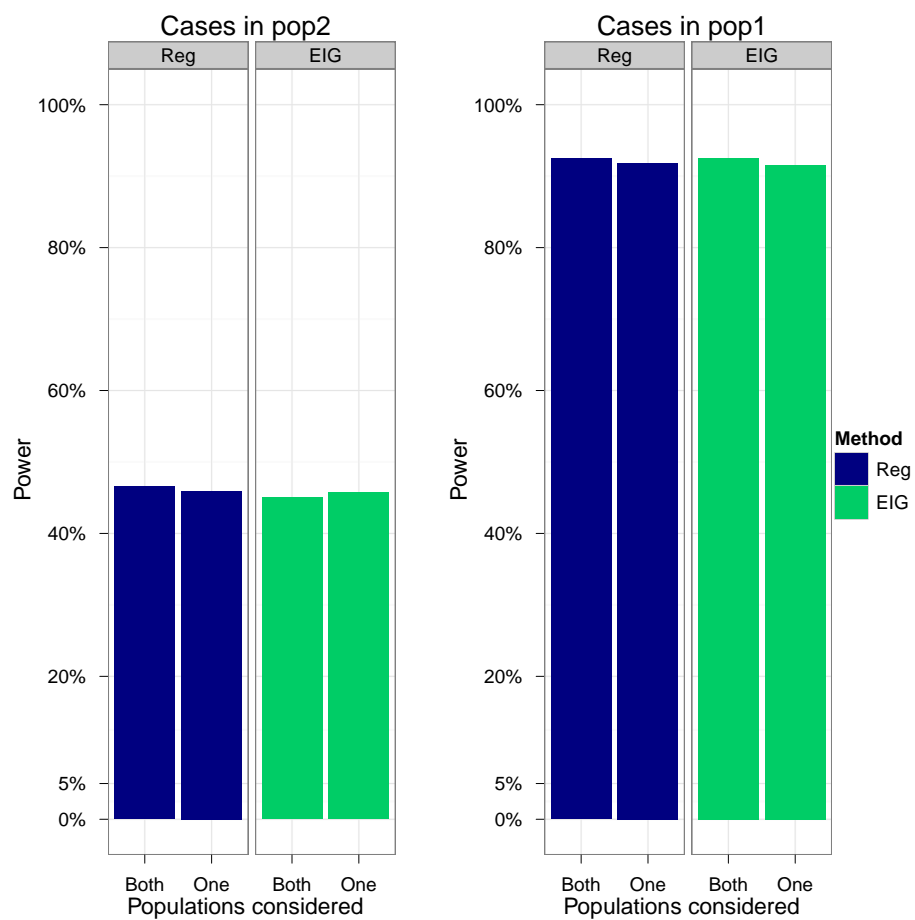

Supplement: Figure S2 — Power comparison for scenario 6 with one or two cohorts. The powers of Reg and Eigenstrat (EIG) are represented when keeping the two populations (Both) or when excluding the population with only controls (One). On the left hand all the cases are in pop2 (less affected by the disease) and on the right hand all the cases are in pop1 (more affected by the disease). (PDF) [file pone.0028845.s004.pdf]

# A – False positive rate comparison

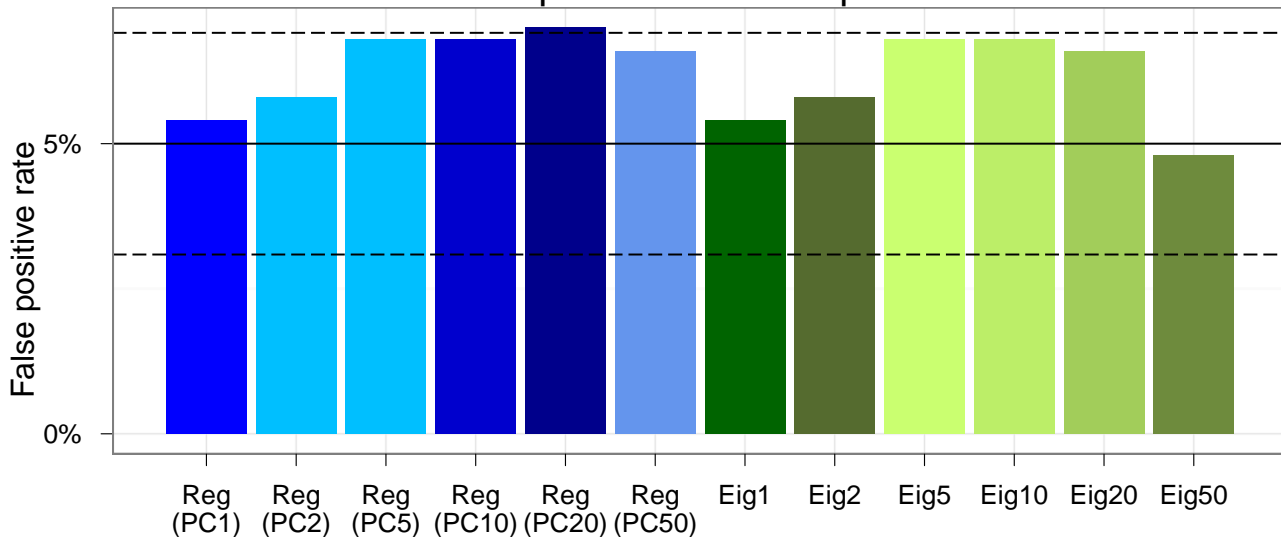

# B – Power comparison

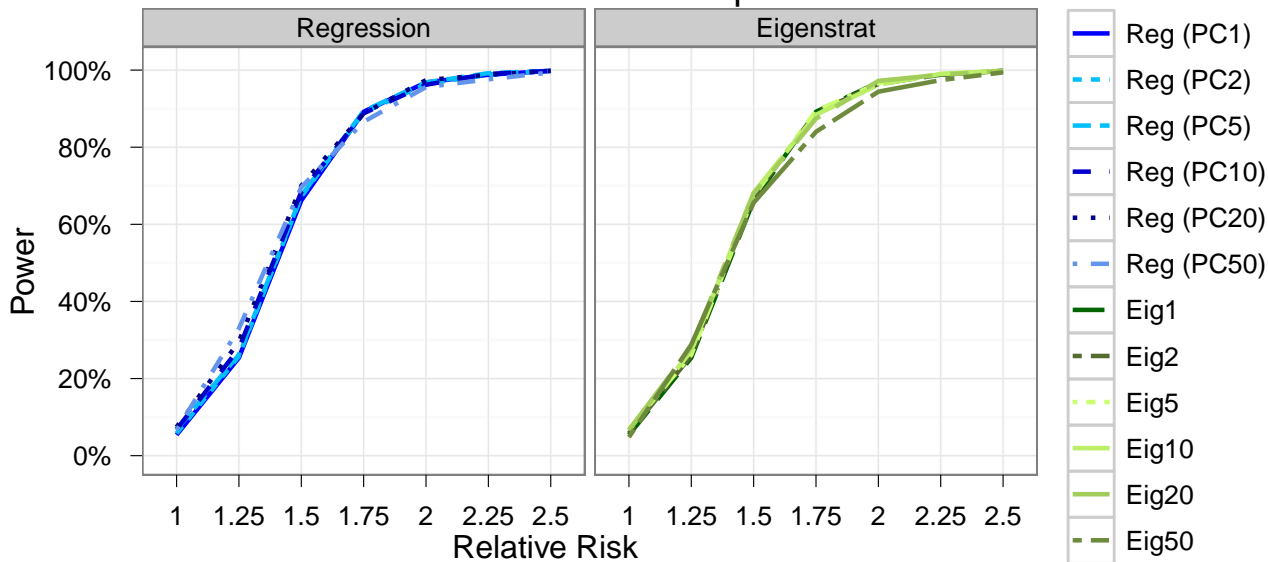

Supplement: Figure S3 — False positive rate and power comparison of principal component based methods with varying number of components included in the models in scenario 4. (PDF) [file pone.0028845.s005.pdf]

# A – False positive rate comparison

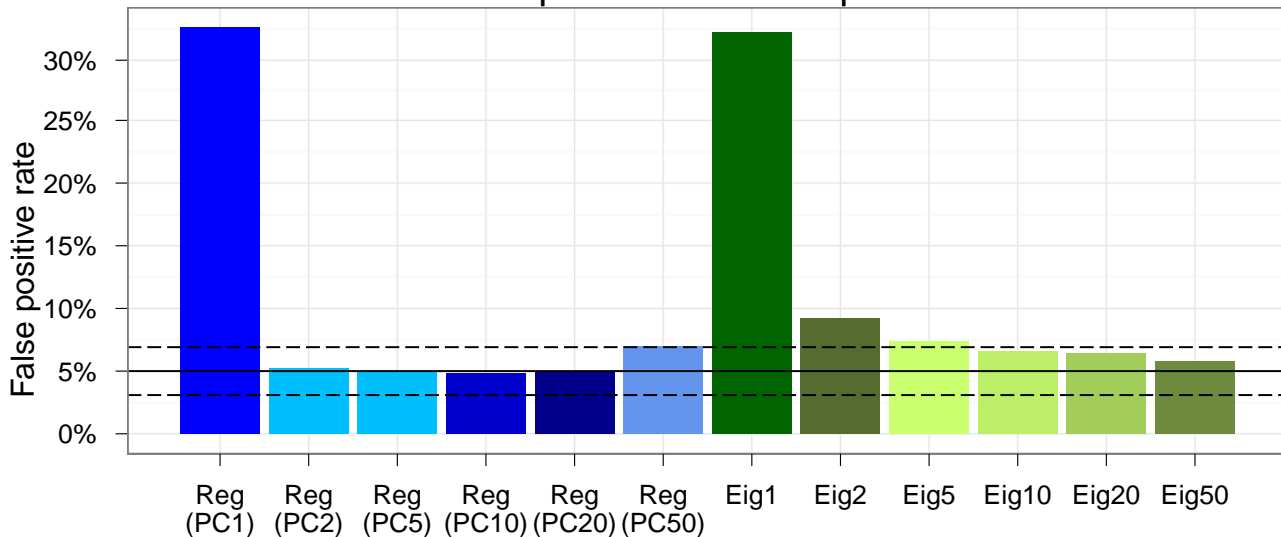

# B – Power comparison

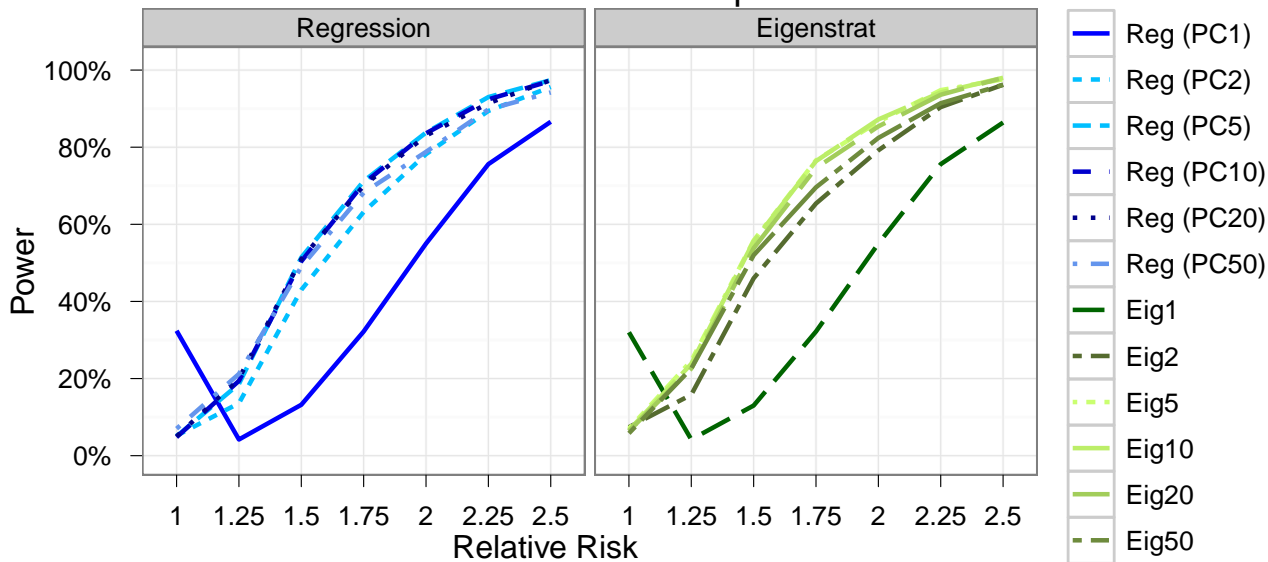

Supplement: Figure S4 — False positive rate and power comparison of principal component based methods with varying number of components included in the models in scenario 5. (PDF) [file pone.0028845.s006.pdf]

### A – False positive rate comparison

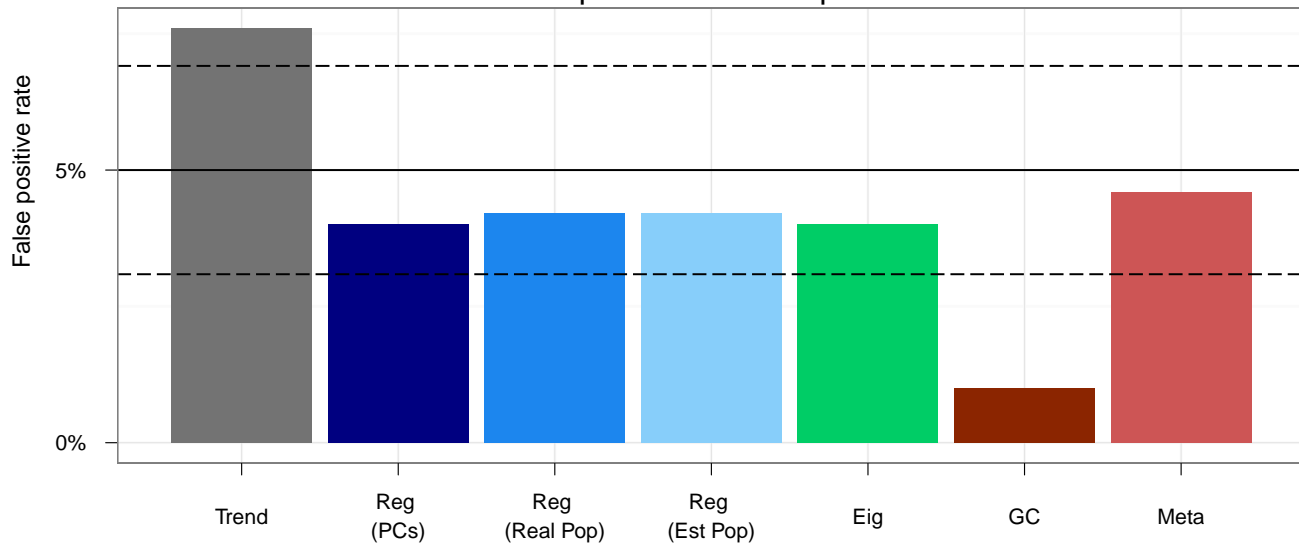

### B – Power comparison

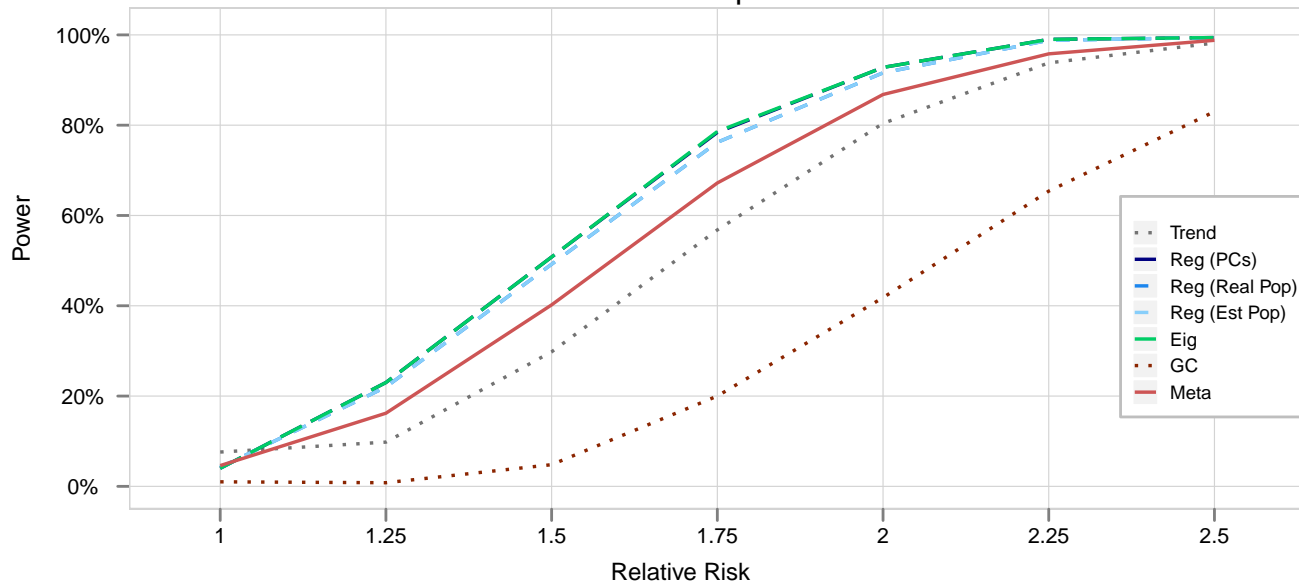

Supplement: Figure S5 — False positive rate and power comparison of the methods with low minor allele frequency for the scenario 4. (PDF) [file pone.0028845.s007.pdf]

### A – False positive rate comparison

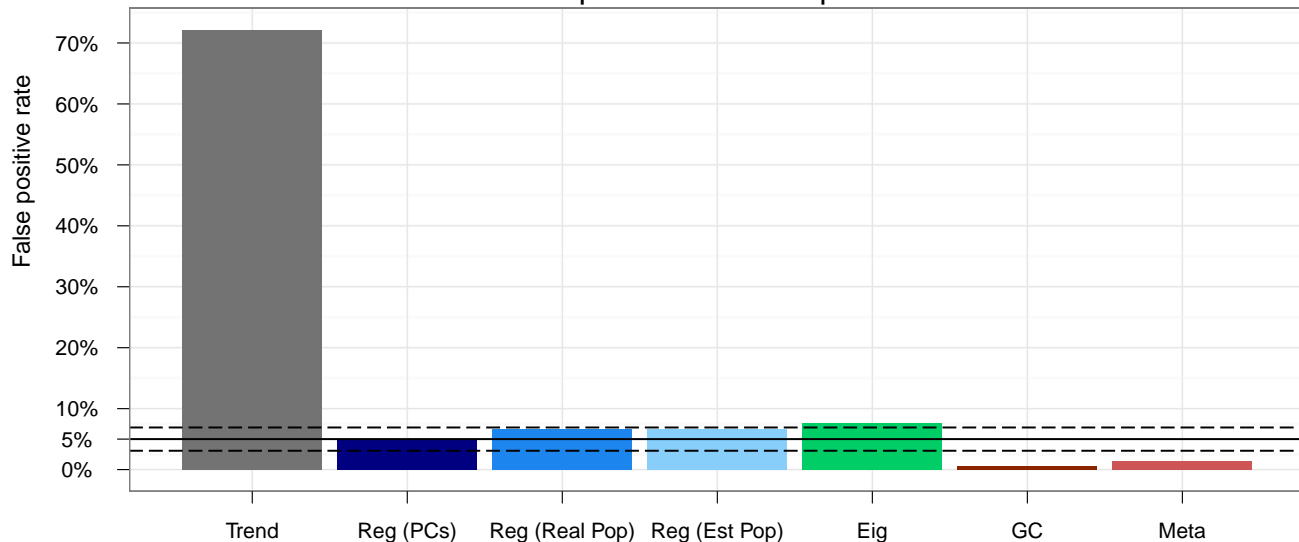

### B – Power comparison

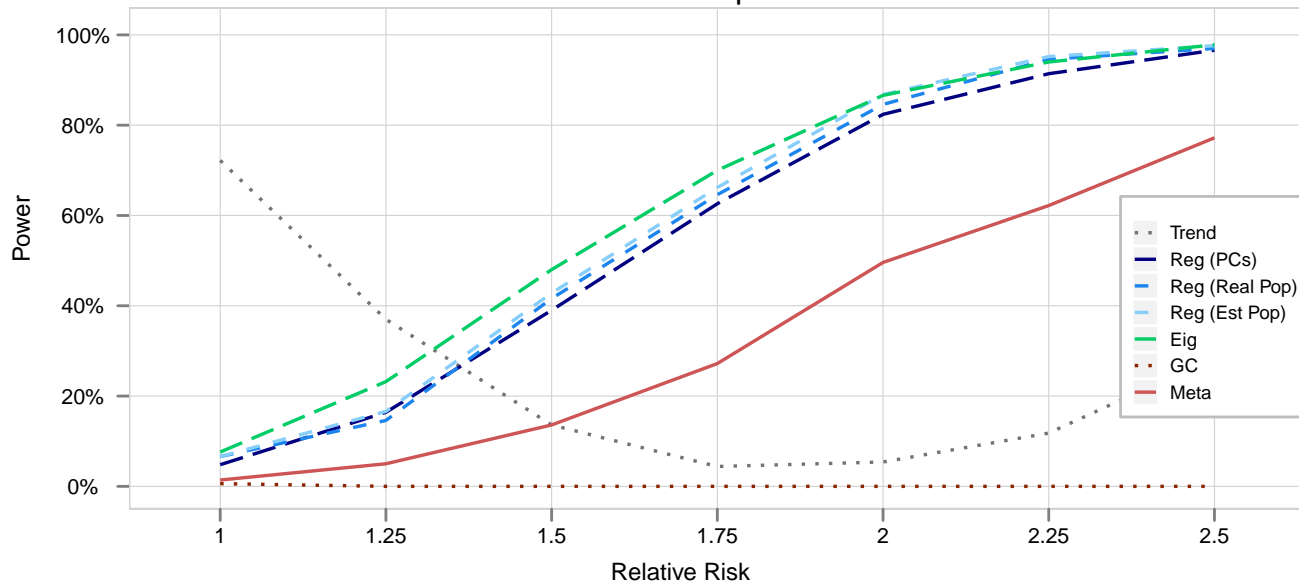

Supplement: Figure S6 — False positive rate and power comparison of the methods with low minor allele frequency for the scenario 5. (PDF) [file pone.0028845.s008.pdf]
