# Supplementary material for: Accounting for Population Stratification in Practice: A Comparison of the Main Strategies Dedicated to Genome-Wide Association Studies
Source: PLoS One. 2011 Dec 21;6(12):e28845. doi: 10.1371/journal.pone.0028845 (PMC3244428; doi:10.1371/journal.pone.0028845)
Supplement: Table S1 — Simulation parameters for the stratification scenarios. (PDF) [file pone.0028845.s009.pdf]

Scenario (Structure)	Populations	Prevalence (K_p)	p_a	Cases	Controls
1 (None)	CHB	0.05	0.3	200	200
2 (Admixture)	CHD	0.05	0.3	125	75
	CHB	0.01	0.2	75	125
3 (Discrete)	CEU	0.05	0.3	125	75
	TSI	0.01	0.2	75	125
4 (Discrete)	CHB	0.05	0.3	125	75
	CEU	0.01	0.2	75	125
5 (Hierarchical)	GIH	0.05	0.3	10	60
	LWK	0.01	0.4	30	50
	YRI	0.01	0.4	30	50
	CEU	0.05	0.2	10	60
	CHB	0.03	0.1	150	10
6 (Discrete)	CHB	0.05	0.3	200.r	100
	CEU	0.01	0.2	200.(1-r)	100
