# Supplementary material for: Accounting for Population Stratification in Practice: A Comparison of the Main Strategies Dedicated to Genome-Wide Association Studies
Source: PLoS One. 2011 Dec 21;6(12):e28845. doi: 10.1371/journal.pone.0028845 (PMC3244428; doi:10.1371/journal.pone.0028845)
Supplement: Table S2 — Estimated for the different scenarios. (PDF) [file pone.0028845.s010.pdf]

	λ
Scenario 1	1.002
Scenario 2	1.009
Scenario 3	1.065
Scenario 4	2.711
Scenario 5	9.571
